# Comparing Two Approaches for Thyroidectomy: A Health Technology Assessment through DMAIC Cycle

**DOI:** 10.3390/healthcare10010124

**Published:** 2022-01-08

**Authors:** Carlo Ricciardi, Adelmo Gubitosi, Donatella Vecchione, Giuseppe Cesarelli, Francesco De Nola, Roberto Ruggiero, Ludovico Docimo, Giovanni Improta

**Affiliations:** 1 Department of Electrical Engineering and Information Technology, University of Naples “Federico II”, 80125 Naples, Campania, Italy; carloricciardi.93@gmail.com; 2 Bioengineering Unit, Institute of Care and Scientific Research Maugeri, 82037 Telese Terme, Campania, Italy; 3 Plastic Surgery, Multidisciplinary Department of Medical Surgical and Dental Specialities, University of Campania “Luigi Vanvitelli”, 80138 Naples, Campania, Italy; adelmo.gubitosi@unicampania.it; 4 Teoresi S. p. A.–Via F. Imparato, 198 CM2, 80146 Napoli, Campania, Italy; donatella.vecchione@teoresigroup.com (D.V.); francesco.denola@teoresigroup.com (F.D.N.); 5 Department of Chemical, Materials and Production Engineering, University of Naples “Federico II”, 80125 Naples, Campania, Italy; 6 Division of General, Mininvasive and Bariatric Surgery, University of Campania “Luigi Vanvitelli”, 80138 Naples, Campania, Italy; roberto.ruggiero@unicampania.it (R.R.); ludovico.docimo@unicampania.it (L.D.); 7 Department of Public Health, University of Naples “Federico II”, 80131 Naples, Campania, Italy; ing.improta@gmail.com; 8 Interdipartimental Center for Research in Healthcare Management and Innovation in Healthcare (CIRMIS), University of Naples “Federico II”, 80131 Naples, Campania, Italy

**Keywords:** health technology assessment, Six Sigma, DMAIC, thyroidectomy

## Abstract

Total thyroidectomy is very common in endocrine surgery and the haemostasis can be obtained in different ways across surgery; recently, some devices have been developed to support this surgical phase. In this paper, a health technology assessment is conducted through the define, measure, analyse, improve, and control cycle of the Six Sigma methodology to compare traditional total thyroidectomy with the surgical operation performed through a new device in an overall population of 104 patients. Length of hospital stay, drain output, and time for surgery were considered the critical to qualities in order to compare the surgical approaches which can be considered equal regarding the organizational, ethical, and security impact. Statistical tests (Kolmogorov–Smirnov, *t* test, ANOVA, Mann–Whitney, and Kruskal–Wallis tests) and visual management diagrams were employed to compare the approaches, but no statistically significant difference was found between them. Considering these results, this study shows that the introduction of the device to perform total thyroidectomy does not guarantee appreciable clinical advantages. A cost analysis to quantify the economic impact of the device into the practice could be a future development. Healthy policy leaders and clinicians who are requested to make decisions regarding the supply of biomedical technologies could benefit from this research.

## 1. Introduction

Total thyroidectomy (TT) is the most performed operation in endocrine surgery, representing the most appropriate therapeutic option in many thyroid disorders [1]. Thyroid is a richly vascularized organ, so the haemostasis is a priority to avoid hard complications [2]. An exhaustive haemostasis makes it possible to prevent potential lesions of the parathyroid glands with consequential hypoparathyroidism, damage of the laryngeal nerves and dangerous post-operative bleeding. Haemostasis obtained with traditional methods, such as clamp and tie, use of clips, electrocautery, or fibrin glue, is time consuming and carries the risk of knot slipping, dislodgment, and thermal damage [3].

At date, the methods allowing medical staff to conduct intervention are the following three:

- TT (or conventional thyroidectomy), which involves a single surgical incision, of discrete size, in the centre of the neck, exactly in correspondence with the thyroid (invasive approach) [4].
- Endoscopic (or minimally invasive) thyroidectomy, which involves two very small incisions on the neck, the use of an endoscope, equipped with a camera and connected to an external monitor, and a surgical instrument for removing the thyroid [5].
- Robotic trans-axillary thyroidectomy by means of 7–8 cm incisions made in the armpits (the neck is not touched), through which the surgeon, using a sort of robotic arms and a camera connected to an external monitor, practices the removal of the thyroid gland [6].

Recently, innovative vessel sealing devices have been recommended in order to give a valuable contribution in terms of accuracy of haemostasis, reducing of operative time and postoperative pain, safety, knowing that the reduction of surgical times is a necessity in terms of cost effectiveness [7]. The Harmonic scalpel (“Focus” and the new version “Focus+”) is one of the first devices for surgical simultaneous cutting and tissue coagulation, which allows to obtain dissection and haemostasis by direct application of ultrasound and allows minimally invasive surgical procedures with minimal lateral thermal spread and, thus, minimal adjacent tissue destruction [8]. The bipolar and radiofrequency sealing system (Ligasure Small Jaw) is an electrosurgical radiofrequency device with haemostatic mechanism, causing a biologic seal which tightly closes the vessels. In a recent single centre register base study evaluating 3346 patients, Ligasure Small Jaws was associated with a reduced risk of postoperative haemorrhage and less postoperative drainage [9]. Another device is the hybrid ultrasonic advanced bipolar (Hub) that integrates ultrasonic energy and advanced bipolar energy, but there was no significant difference with the ultrasonic device in postoperative surgical results and morbidity [10]. Transoral endoscopic thyroidectomy vestibular approach grows in popularity and there is a need for data on cost in order to better characterize its value to patients [11].

Furthermore, post-operative complications cannot be neglected especially in function of the possible consequences on the patients’ health” [12]. These complications are divided into:

- Hypocalcaemia due to “hypoparathyroidism”, which develops following the removal of the entire thyroid and sees a temporary inactivity of the parathyroid glands, which normally regresses spontaneously or with calcium and vitamin D therapies [13,14];
- Dysphonia, due to the lesion of the recurrent laryngeal nerve (which controls the movement of the vocal cords), which is in close contact with the thyroid gland and is extremely sensitive to minor trauma, strains, and inflammation, which are a normal consequence of surgery [15];
- Post-operative bleeding due to the particular vascular network of gland treated by surgical drainage [16,17,18,19,20];
- Bacterial infection in the operated area;
- Airway obstruction due to prolonged bleeding.

### An introduction to the Methodology

In this scenario, different managerial approaches could simulate and improve the process. Among them, Health Technology Assessment (HTA) [21] and Six Sigma (SS) [22] proved to be reliable and promising approaches in the healthcare context [23,24].

HTA provides easy-to-use tools to decision makers through scientific and rigorous methods. It is based on the development of safe, effective, patient-focused health policies and seeks to add value, as defined by decision makers [25]. Its functioning is related to the organization of a multidisciplinary team. It can be composed of a broad variety of professionals (e.g., physicians, nurses, managers of healthcare institute, laboratory technicians, patients, epidemiologists, economics, lawyers, and clinical and biomedical engineers) depending upon the scope of the assessment [26].

This concept is based on effectiveness, efficiency, and equity intervention [27], as also established by the World Health Organization [28].

On the other side, SS is defined by Linderman et al. as “(…) an organized and systematic method for strategic process improvement and new product and service development that relies on statistical methods and the scientific method to make dramatic reductions in customer defined defect rates” [29]. Indeed Lean Six Sigma (LSS) is a managerial concept that combines the lean manufacturing philosophy [30] of the effectiveness and the incorporation of the SS quality management program [31].

This program should be considered as a new engineering approach to producing more responsive supply chains through effective communication, strategic alliances, and visibility [32]. Indeed, SS is a method that has combined the most successful aspects of previous approaches and that has the ability to improve their organization in the most powerful tools [33]. Lean Management provides the opportunities of reducing the costs and cycle time, eliminating unnecessary passages [34]. The implementation of Lean Management and SS techniques allows a better clarification of each value adding step and on the other hand it fixes the problems of the flow of activities and the activities that do not add value, and it provides a second entry to Lean Manufacturing and management techniques [35].

In the last decades, the LSS was implemented as an HTA tool, especially for the part concerning the production and processing of data to support the decision-making process, through the implementation of the 5-phase procedure of SS called DMAIC [36].

Originally described as a method for variation reduction, DMAIC is applied in practice as a generic problem solving and improvement approach [37]. DMAIC helps to identify the root causes of the problem and define the control measures for the same.

The aim of this work is to carry out an HTA procedure to compare two different surgical procedures using an adapted DMAIC cycle of SS, as recently performed in literature [38,39]. The rationale is to produce helpful data for the evaluation of the best surgical technique, and therefore, more generally, for the decision-making and management processes of health governance. The two surgical procedures are different versions of the same surgical operation of TT i.e., total removal of the thyroid: the procedure defined as traditional involves, after the first phase common to the two incisions in the lower part of the neck, the cutting and haemostasis of highly vascularized tissues with sutures and/or carbonization or denaturation of proteins; the procedure with a device, on the other hand, uses pressure and radiofrequency waves to simultaneously perform tissue sectioning and haemostasis by synthesizing the tissue wall by fusion of elastin and collagen naturally present within the blood vessels.

The processes and characteristics of HTA were combined with those of the SS, taking the DMAIC cycle from the latter, to analyse the available data and obtain ad hoc information that can help decision makers in the process evolution of the healthcare system. In particular, traditional TT was compared with TT performed through a device considering seven relevant variables as input and three outcomes: length of hospital stay (LOS) (which was already used previously in literature [38,40]), drain output (which was recently employed for similar scopes in literature [39,41]) and surgery duration. We can consider the big picture of this work as an HTA in order to evaluate the usefulness of purchasing specific devices for the surgical activity in detail in order to reduce the patient’s stay time in the surgical facility and improve the clinical conditions after surgery compared to traditional surgery methods.

## 2. Materials and Methods

### 2.1. Integrating DMAIC Cycle into HTA

DMAIC roadmap is an evolution of the Plan-Do-Check-Act strategy consisting in a data-driven, systematic, and fact-based cycle usually included in SS; they have both been employed for the continuous improvement of processes [42]. The key difference between them is that the former is led by data (data-driven) and used for improving, empowering, and stabilizing business processes while the latter is a repetitive model made up of 4 phases.

The 5 phases of DMAIC are, usually, the following [43,44,45,46]:

1. Define aims at defining the project, the issues, and the scopes.
2. Measure aims at measuring quantitatively the critical to quality (CTQ) of the current process.
3. Analyse aims at conducting statistical analysis to examine the causes and effects of the inefficiencies in the current process or the variables influencing it.
4. Improve aims at introducing some corrective actions to improve the process or, in an HTA context, at comparing a couple of biomedical technologies or clinical pathways.
5. Control aims at guaranteeing long-rung results and employs statistical analyses to confirm the improvement.

Because SS provides policy makers with a systematic and quantitative scheme through the DMAIC cycle, which is generalizable for an extensive variety of applications, it can represent a valuable support tool for HTA [24,38], providing comprehensive analysis, evidence-based decisions, and efficient control plan. Indeed, several studies of HTA have employed SS as a rigorous methodology to compare biomedical technologies and understand which can be considered the best according to some parameters defined according to the specific analysed domain [40,46].

In this paper, the DMAIC cycle was used to compare the thyroidectomy performed with a new device with the traditional one; define, measure, and analyse phases were strictly followed while the improve phase was replaced by the description of the new procedures and the control phase consisted in identifying the statistical tests propaedeutic to conduct the statistical comparison. The DMAIC strategy has been implemented in this research, as it follows.

### 2.2. Define

In the define phase, the team dealing with the project was defined and contained biomedical and management engineers, clinicians, and surgeons. A brief project charter was written to identify the main parts of the project:

- Project title: HTA to evaluate the introduction of a device to perform thyroidectomy.
- CTQ: LOS, drain output, and time for surgery.
- In scope: Thyroidectomy. University of Campania “Luigi Vanvitelli”, Division of General Surgery, Department of Surgery.
- Out of scope: All the other structures and interventions.
- Financial: No funding to reach the target.
- Business need: Evaluating the usefulness of the device introduced for thyroidectomy according to the CTQs.

The aim of the project was basically the implementation of an HTA study by using the DMAIC strategy as already performed in previous research, where the DMAIC cycle and SS have shown their feasibility [38,39,41]; in particular, the surgical procedures were compared according to the CTQs previously defined.

### 2.3. Measure

After defining the main part of the project in the define phase, in the measure phase the starting datasets were characterized with descriptive statistics (Table 1) and visual management tools such as simple histograms for both groups (traditional surgery and surgery with the device). The data were collected during the thyroidectomy course, which requires an average of three days of hospital stay. The dataset was divided into two parts: Group A and Group B. Group A includes patients who underwent surgery through the use of the device while patients in group B are those who underwent surgery through traditional surgery.

In group A (device surgery group), the patients (*n* = 52) were treated by using the device while in group B (traditional surgery group) (*n* = 52), patients received thyroidectomy through the device. Patients were afferent to the Department of Surgery, University of Campania “Luigi Vanvitelli”.

The mean age was 55.62 ± 12.68years in group A and 53.15 ± 13.51 years in group B (*p*-value = 0.347); in the former group, the mean Body Mass Index (BMI) was 27.52 ± 6.19 while for the latter one it was 26.23 ± 4.28 (*p*-value = 0.229). The distribution of patients according to gender was almost the same (*p*-value = 0.813). The characteristics are summarized in Table 1.

The two datasets were compared through a Mann–Whitney test and a chi square for the gender variable and can be considered homogeneous in regards to age, gender, and BMI.

### 2.4. Analyse

In the analysis phase, statistical analyses were conducted to identify the variables contributing substantially to the CTQs, as performed previously in literature [31,36,42]. Indeed, all the variables were analysed in order to understand which one could influence the CTQ for both groups. A normality test was performed (*p* < 0.001); due to the non-normality distributions of the data, Mann–Whitney tests were performed with an uncertainty level of 0.05.

The tables with the related results are shown in the section “Results”.

### 2.5. Improve: Traditional Surgery and Thyroidectomy through the Device

The conventional thyroidectomy surgery is applied for the complete removal of the thyroid gland; it involves a skin incision at the base of the neck in the anterior position, generally practiced two fingers above the jugular dimple (“neck cervicectomy according to Kocher”). Then, it is necessary to proceed to the preparation and suspension of the upper musculocutaneous flap by blunt way on the platysma, to the section of the median raphe, retraction of the pre-thyroid muscles, exposure and extracapsular dissection of the thyroid with interruption of the vascular peduncles (which can be sectioned between ligatures or metal clips, or coagulated with haemostatic devices), and preservation of the parathyroid glands (usually two on each side) and the lower or recurrent laryngeal nerves (one on each side). The operation ends with the reconstruction of the muscle plane (sternohyoid and sternothyroid muscle) and with the suture of the platysma muscle, together with the subcutaneous plane and the skin. Depending on the case, one or two drains are positioned, sometimes in suction, laterally, or inferiorly to the wound, to facilitate the attachment of the surfaces and to remove any serous and blood secretions. The drain is usually removed after 48 h. However, there are serious pathological conditions for which it is necessary to enlarge the operative field with a median sternotomy.

#### The New Development Regarding Device for Thyroidectomy

Recently, innovative vessel sealing devices have been recommended in order to give a valuable contribution in terms of accuracy of haemostasis, reducing of operative time and postoperative pain, safety, knowing that the reduction of surgical times is a necessity in terms of cost effectiveness. The ultrasonically activated shear (Focus) is among the first devices for surgical simultaneous cutting and tissue coagulation, which allows to obtain dissection and haemostasis by direct application of ultrasound and allows minimally invasive surgical procedures with minimal lateral thermal spread and, thus, minimal adjacent tissue destruction. Ultrasonic shear is a really innovative device for many aspects, and it is already widely used in laparoscopic surgery, implying surgical time-saving and low incidence of complication rates. The first reference about the use of the ultrasonic scalpel in thyroid surgery dates to 2000. Then numerous studies have confirmed the validity of this innovative technology. Moreover, during the last ten years, on the based experience of the surgeons who used the ultrasonic scalpel, both ergonomic and technical changes were made to improve the dissection and haemostasis of tissue.

The bipolar radiofrequency sealing system (Ligasure Small Jaw) is an electrosurgical radiofrequency device with a haemostatic mechanism causing a biologic seal which tightly closes the vessels. It is a disposable instrument generally indicated in neck surgery [47] that simultaneously allows tissue section and vessels haemostasis. It consists of an ergonomic handle and a terminal forceps, combining the forceps pressure and radiofrequency applied to the tissues target [48]. Indeed, the tissue dissection is performed thought the heat-controlled radiofrequency current. Moreover, haemostasis is achieved through the fusion of collagen and the elastin of the intimal part of the vessel [49], also near delicate anatomical structures, where thermal diffusion could damage nerve structures [50]. The result is an effective change in the nature of the vessel walls in which collagen and elastin merge to create a tissue identical to the original one [51].

Surgical technique requires the Minimally Invasive Thyroidectomy approach (3–5 cm incision) in presence of a nodule between 35 and 50 mm, or a thyroid total volume between 30 and 80 mL, or both. In presence of thyroid volumes greater than 80 mL, or a thyroid nodule larger than 50 mm, or both, high risk carcinoma TT performed through incision greater than 5.5 cm was indicated. After division of the platysma, the cervical line alba is opened without division of the strap muscles. The thyroid lobe is dissected progressively from the strap muscles. After identification of the recurrent laryngeal nerve and parathyroid glands, the vascular pedicles of the thyroid lobe are ligated with the Focus and Small Jaw and the thyroid lobe is removed. After a check for haemostasis, a drain is always placed in the thyroid bed as part of the study protocol, in order to quantify blood loss during the first 24 h. The cervical line alba and platysma are sutured with absorbable sutures and the skin is closed by an intracutaneous running suture.

Therefore, we compared two surgical procedures of TT performed by using traditional instrumentation and the recent introduction of a technological “Device”.

Given the existence of an identical clinical path for both types of surgical procedures, our analysis focused on the clinical evidence related to the functioning of the “Device”. Indeed, we also analysed the operation physics of the “Device” by comparing it to that of some of its competitors used for the surgical procedure.

“Focus and Small Jaw” confines its effect to the tissue or vessel without charring, and with minimal thermal diffusion to adjacent tissues. The latter represents an evolution of the classic haemostasis method with suturing, or cauterization or carbonization of the damaged tissue, or a combination thereof. Thus, we focused on trying to obtain clinical evidence of the possible superiority of the procedure with the use of the technological tool from the data of the procedure with the device [48,49,50,51].

## 3. Results (Control)

Thus, we identified the best reasonable parameters for Hospitalization, Drainage, and Duration of intervention.

We organized data in categories: age of the patients, sex, Body Mass Index (BMI), thyroid pathology type, state at the time of admission (distinguished between Basedow and Multinodular goiter), presence or absence of previous pathologies such as hypertension, use of anticoagulant drugs, and appearance of post-operative complications such as hypocalcaemia.

Then, we divided the population in two subcategories: patients who have undergone thyroidectomy with the traditional procedure and those who underwent surgery through the device previously described.

We applied the Kolmogorov–Smirnov test to identify if the data distributions were normal, then we performed T and ANOVA tests, for non-normal distributions Mann–Whitney and Kruskal–Wallis tests (depending on whether the population was organized in dichotomous categories or no).

Through the study of the clinical process of patients subjected to thyroidectomy, we defined a scheme to evaluate the hospital path in both cases of “Traditional” and “Device” intervention. It was divided in:

- Pre-hospitalization.
- Recovery.
- Surgical procedure.
- Discharge or prolonged post-operative hospitalization due to complications.

The qualitative survey was conducted for distinguishing patients undergoing traditional intervention and those who had undergone a procedure with the device. Thus, it was possible to identify two subpopulations divided into “Traditional” and “Device” and analyse their individual characteristics.

**Figure 1:**
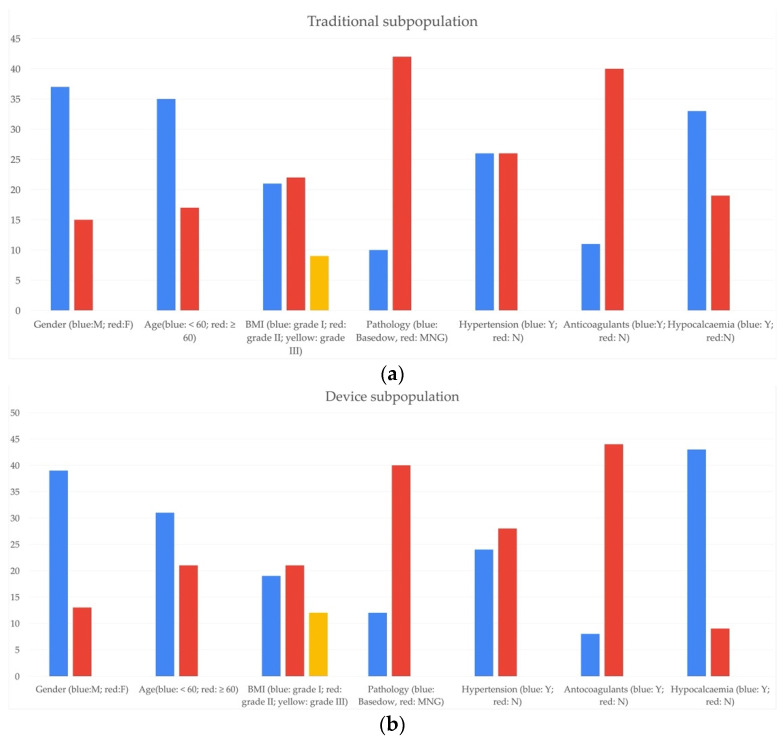
Demographic survey of (**a**) “Traditional” subpopulation and (**b**) “Device” subpopulation. In both figures, the y axis shows the number of patients.

The demographic survey of the “Traditional” and “Device” subpopulation led to the composition of the following graphs (Figure 1).

In accordance with the epidemiological data, a significant majority of female patients are visible, a consistent presence of patients with multinodular goitre (among the thyroid pathologies of interest) in both subpopulations, while age difference seems to be less relevant for “Device” subpopulation.

**Figure 2:**
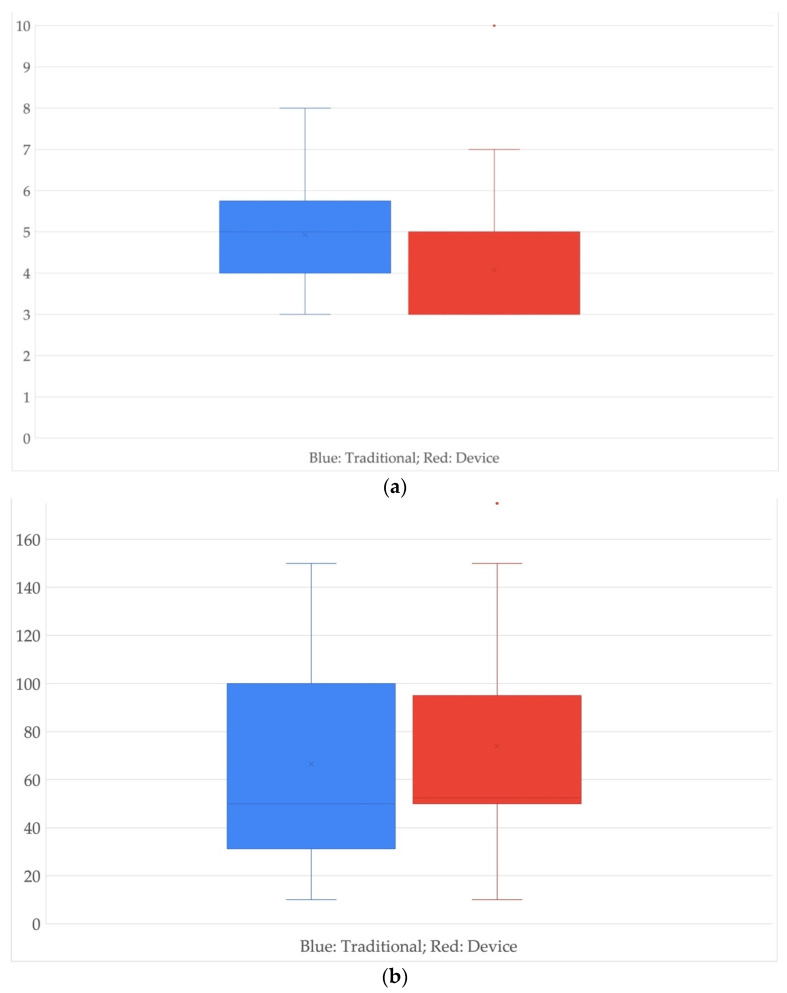
Boxplot to compare (**a**) LOS “Traditional vs. Device” (y axis unit: days), (**b**) drain output “Traditional vs. Device” (y axis unit: c.c.).

The following boxplots (Figure 2) will provide additional information; first, in the left boxplot—see Figure 2a—the average is of four days for the first case and three days for the second.

In the second boxplot—see Figure 2b—the average for patients undergoing a traditional surgical procedure compared with those undergoing devices is identical for both cases and is around 50 c.c. of medium drained blood.

After the use of visual management tools, as per SS methodology, the assessment was performed with a statistical approach. Table 2, Table 3, Table 4, Table 5, Table 6 and Table 7 represent the analysis phase of the DMAIC cycle where the influence on the CTQs from the variables included in the research is investigated.

Table 2 and Table 3 show that gender and pathology are likely to influence the LOS in a statistically significant way while there is no statistical evidence of a particular influence from the variables on the drain output of patients or surgery duration. Table 6 and Table 7 do not include hypocalcaemia because it emerges after the intervention, thus it cannot interfere with surgery duration.

Afterward a comparison between “traditional” and “device” group is performed according to the three CTQs: LOS, drain output, and surgery duration (Table 8, Table 9 and Table 10).

Table 8, Table 9 and Table 10 show that there is no evidence of any improvement in LOS, drain output, or surgery duration according to any of the variables after the introduction of the device for TT in the analysed groups of patients.

## 4. Discussion

The use of DMAIC cycle for assessing two biomedical technologies or medical processes has recently been appreciated in literature [38,39,40] but it has again shown its usefulness for guiding researchers into the solution of HTA.

In this study, we showed that “Hospitalization” is influenced by the pathology variables and the onset of hypocalcaemia in patients undergoing traditional surgery; moreover, because the variables gender and hypertension are just not less than 5%, but still very low, they can be considered possibly relevant. The first two variables certainly influence the hospitalization in the case of TT, while the remaining two, at date, do not seem to influence it. For patients undergoing a procedure with a “Device”, hospitalization seems to be influenced by two variables: gender and pathology.

We repeated the same type of examination for the “Drainage”, detecting only a not statistically significant value for the hypocalcaemia variable in the “Device” case.

The onset of the complication of hypocalcaemia probably affects the volume of blood drained (or more probably it is the volume of blood lost by the patient that affects the risk of occurrence or not of this complication). This result is linked to the functioning of the instrument of the haemostasis phase because, according to the physics of the functioning of the instrument used, the “device” technique would allow a less negative impact.

Regarding the “Duration” of the surgery, none of the variables available are statistically significant: none of these variables influences our CTQs; therefore, the time taken to perform the surgical procedure and this conclusion is verified for both surgical procedures.

Focusing on all statistically significant variables comparing patients who have undergone “Traditional” and “Device” thyroidectomy, we identified the conditions for the best procedure.

“Hospitalization” variables were slightly higher than 5% (without ever being statistically significant) in the case of surgical “device” and it could not be considered absolutely as the best procedure. Unless further analysis, the hospitalization of obese patients, without previous pathologies (hypertension) and who do not take anticoagulants could be slightly better (shorter) in the case of a surgical procedure with “Device”.

“Drainage” Basedow pathology approaches have a value of 5%, without however being “statistically significant”. This could mean, without having statistical confirmation, that for patients with Basedow, there is the possibility that the use of the device may cause a decrease in the volume of blood lost by the patient.

There are no statistically significant values for any of the variables processed applied for “Duration” of the surgery.

For “Drainage”, both in the traditional and device case, gender and pathology were statistically significant variables while for “Duration” of surgery, gender, pathology, and hypertension were relevant in both cases.

## 5. Conclusions

According to this study, the device does not introduce a statistically significant improvement for TT as regards LOS, drain output, or surgery duration. The information provided in this paper could be useful for healthy policy leaders and for clinicians who are requested to make decision regarding the supply of biomedical technologies. Of course, further studies could focus on cost analysis in order to also quantify the economic impact of introducing the device into the clinical practice.

The study of course has some limitations; there might be some other variables that could be considered, although we considered seven variables as input, which are surely sufficient in this context, and three robust CTQs in this analysis. In the end, the work developed can be used by exploiting the same quantities and the same data processing to view any variations and applications for the specific case for the different types of devices used in clinical practice.

## Figures and Tables

**Table 1:** Descriptive statistics of the population. Mann–Whitney = ’, Chi square = ^. Abbreviation: BMI: Body Mass Index.

Category	Device *n* = 52	Traditional *n* = 52	*p*-Value
Age (years)	55.62 ± 12.68	53.15 ± 13.51	0.347 ’
Gender (males/females)	11/41	12/40	0.813 ^
BMI	27.52 ± 6.19	26.23 ± 4.28	0.229 ’

**Table 2:** Analysing variables influencing LOS for traditional group. Statistically significant results (significance level = 0.05) = *. Abbreviation: BMI: Body Mass Index.

Variables	Categories	Traditional LOS			*p*-Value
		Mean	±	SD	
Gender	Males	3.42	±	0.90	0.052
	Females	4.22	±	1.33	
Age	<60	3.97	±	1.14	0.984
	>60	4.17	±	1.54	
BMI	Normal	4.19	±	1.33	0.244
	Overweight	3.64	±	0.85	
	Obese	4.67	±	1.80	
Pathology	Basedow	3.30	±	0.67	0.027 *
	Multinodular goiter	4.21	±	1.33	
Hypertension	Yes	3.81	±	1.44	0.063
	No	4.27	±	1.08	
Anticoagulants	Yes	3.64	±	1.03	0.297
	No	4.15	±	1.33	
Hypocalcaemia	Yes	3.78	±	1.37	0.029 *
	No	4.33	±	1.13	

**Table 3:** Analysing variables influencing LOS for Device group. Statistically significant results (significance level = 0.05) = *. Abbreviation: BMI: Body Mass Index.

Variables	Categories	Device LOS			*p*-Value
		Mean	±	SD	
Gender	Males	3.18	±	0.60	0.034 *
	Females	3.90	±	1.14	
Age	<60	3.70	±	0.99	0.973
	>60	3.80	±	1.24	
BMI	Normal	4.05	±	1.27	0.268
	Overweight	3.60	±	0.99	
	Obese	3.45	±	0.82	
Pathology	Basedow	3.00	±	0.00	0.003 *
	Multinodular goiter	3.97	±	1.15	
Hypertension	Yes	3.74	±	1.17	0.797
	No	3.74	±	1.02	
Anticoagulants	Yes	4.28	±	1.70	0.412
	No	3.65	±	0.95	
Hypocalcaemia	Yes	3.59	±	0.95	0.029 *
	No	4.15	±	1.34	

**Table 4:** Analysing variables influencing Drain output for traditional group. Abbreviation: BMI: Body Mass Index.

Variables	Categories	Traditional Drain Output			*p*-Value
		Mean	±	SD	
Gender	Males	52.08	±	40.53	0.165
	Females	70.87	±	43.58	
Age	<60	61.17	±	40.84	0.197
	>60	76.67	±	47.00	
BMI	Normal	69.05	±	43.03	0.755
	Overweight	61.82	±	43.52	
	Obese	72.22	±	47.05	
Pathology	Basedow	52.00	±	15.13	0.520
	Multinodular goiter	70.00	±	47.05	
Hypertension	Yes	65.00	±	40.32	0.904
	No	68.07	±	46.75	
Anticoagulants	Yes	71.36	±	32.41	0.288
	No	65.24	±	45.99	
Hypocalcaemia	Yes	62.68	±	36.88	0.796
	No	71.04	±	50.13	

**Table 5:** Analysing variables influencing Drain output for Device group. Abbreviation: BMI: Body Mass Index.

Variables	Categories	Device Drain Output			*p*-Value
		Mean	±	SD	
Gender	Males	57.73	±	19.54	0.414
	Females	78.29	±	56.18	
Age	<60	76.93	±	53.19	0.843
	>60	69.52	±	49.09	
BMI	Normal	87.63	±	59.71	0.190
	Overweight	54.28	±	22.32	
	Obese	86.67	±	64.68	
Pathology	Basedow	63.75	±	16.67	0.733
	Multinodular goiter	77.00	±	57.53	
Hypertension	Yes	70.62	±	46.98	0.941
	No	76.78	±	55.28	
Anticoagulants	Yes	71.87	±	33.90	0.662
	No	74.32	±	54.06	
Hypocalcaemia	Yes	72.24	±	57.06	0.076
	No	78.57	±	31.46	

**Table 6:** Analysing variables influencing Surgery duration for Traditional group. Abbreviation: BMI: Body Mass Index.

Variables	Categories	Traditional Surgery Duration			*p*-Value
		Mean	±	SD	
Gender	Males	78.75	±	16.53	0.499
	Females	75.00	±	16.56	
Age	<60	76.17	±	15.67	0.861
	>60	75.28	±	18.35	
BMI	Normal	73.57	±	18.45	0.654
	Overweight	61.82	±	43.52	
	Obese	79.44	±	14.67	
Pathology	Basedow	79.50	±	14.03	0.396
	Multinodular goiter	75.00	±	17.03	
Hypertension	Yes	76.54	±	16.54	0.772
	No	75.19	±	16.70	
Anticoagulants	Yes	76.36	±	13.05	0.896

**Table 7:** Analysing variables influencing Surgery duration for Device group. Abbreviation: BMI: Body Mass Index.

Variables	Categories	Device Surgery Duration			*p*-Value
		Mean	±	SD	
Gender	Males	81.82	±	17.07	0.184
	Females	75.61	±	20.01	
Age	<60	76.29	±	15.33	0.729
	>60	77.86	±	24.68	
BMI	Normal	71.05	±	14.49	0.105
	Overweight	54.28	±	22.32	
	Obese	84.17	±	19.05	
Pathology	Basedow	77.08	±	15.29	0.836
	Multinodular goiter	76.87	±	20.68	
Hypertension	Yes	76.25	±	24.90	0.803
	No	77.50	±	13.57	
Anticoagulants	Yes	81.25	±	33.14	1.000
	No	76.14	±	16.28	

**Table 8:** Comparison between “Traditional” and “device” groups according to LOS. Abbreviation: BMI: Body Mass Index.

Variables	Categories	Traditional LOS			Device LOS			*p*-Value
		Mean	±	SD	Mean	±	SD	
All		4.04	±	1.28	3.74	±	1.08	0.179
Gender	Males	3.42	±	0.90	3.18	±	0.60	0.425
	Females	4.22	±	1.33	3.90	±	1.14	0.233
Age	<60	3.97	±	1.14	3.70	±	0.99	0.269
	>60	4.17	±	1.54	3.80	±	1.24	0.413
BMI	Normal	4.19	±	1.33	4.05	±	1.27	0.682
	Overweight	3.64	±	0.85	3.60	±	0.99	0.583
	Obese	4.67	±	1.80	3.45	±	0.82	0.093
Pathology	Basedow	3.30	±	0.67	3.00	±	0.00	0.113
	Multinodular goiter	4.21	±	1.33	3.97	±	1.15	0.386
Hypertension	Yes	3.81	±	1.44	3.74	±	1.17	0.956
	No	4.27	±	1.08	3.74	±	1.02	0.057
Anticoagulants	Yes	3.64	±	1.03	4.28	±	1.70	0.495
	No	4.15	±	1.33	3.65	±	0.95	0.066
Hypocalcaemia	Yes	3.78	±	1.37	3.59	±	0.95	0.797
	No	4.33	±	1.13	4.15	±	1.34	0.489

**Table 9:** Comparison between “Traditional” and “device” groups according to Drain output. Abbreviation: BMI: Body Mass Index.

Variables	Categories	Traditional Drain Output			Device Drain Output			*p*-Value
		Mean	±	SD	Mean	±	SD	
All		66.54	±	43.26	73.94	±	51.21	0.429
Gender	Males	52.08	±	40.53	57.73	±	19.54	0.274
	Females	70.87	±	43.58	78.29	±	56.18	0.688
Age	<60	61.17	±	40.84	76.93	±	53.19	0.230
	>60	76.67	±	47.00	69.52	±	49.09	0.529
BMI	Normal	69.05	±	43.03	87.63	±	59.71	0.373
	Overweight	61.82	±	43.52	54.28	±	22.32	0.922
	Obese	72.22	±	47.05	86.67	±	64.68	0.668
Pathology	Basedow	52.00	±	15.13	63.75	±	16.67	0.099
	Multinodular goiter	70.00	±	47.05	77.00	±	57.53	0.667
Hypertension	Yes	65.00	±	40.32	70.62	±	46.98	0.783
	No	68.07	±	46.75	76.78	±	55.28	0.565
Anticoagulants	Yes	71.36	±	32.41	71.87	±	33.90	0.974
	No	65.24	±	45.99	74.32	±	54.06	0.312
Hypocalcaemia	Yes	62.68	±	36.88	72.24	±	57.06	0.783
	No	71.04	±	50.13	78.57	±	31.46	0.408

**Table 10:** Comparison between “Traditional” and “device” groups according to surgery duration. Abbreviation: BMI: Body Mass Index.

Variables	Categories	Traditional Surgery Duration			Device Surgery Duration			*p*-Value
		Mean	±	SD	Mean	±	SD	
All		75.86	±	16.47	76.92	±	19.43	0.878
Gender	Males	78.85	±	16.53	81.82	±	17.07	0.666
	Females	75.00	±	16.56	75.61	±	20.01	0.909
Age	<60	76.17	±	15.67	76.29	±	15.33	0.976
	>60	75.28	±	18.35	77.86	±	24.68	0.734
BMI	Normal	73.57	±	18.45	71.05	±	14.49	0.632
	Overweight	61.82	±	43.52	54.28	±	22.32	0.980
	Obese	79.44	±	14.67	84.17	±	19.05	0.529
Pathology	Basedow	79.50	±	14.03	77.08	±	15.29	0.704
	Multinodular goiter	75.00	±	17.03	76.87	±	20.68	0.801
Hypertension	Yes	76.54	±	16.54	76.25	±	24.90	0.777
	No	75.19	±	16.70	77.50	±	13.57	0.582
Anticoagulants	Yes	76.36	±	13.05	81.25	±	33.14	0.901
	No	75.73	±	17.41	76.14	±	16.28	0.912
Hypocalcaemia	Yes	77.32	±	15.60	77.37	±	15.71	0.990
	No	74.17	±	17.61	75.71	±	27.86	0.796

## Data Availability

The data presented in this study are available on request from the author R.R. The data are not publicly available due to privacy reasons.
